# The Application of Medical Artificial Intelligence Technology in Rural Areas of Developing Countries

**DOI:** 10.1089/heq.2018.0037

**Published:** 2018-08-01

**Authors:** Jonathan Guo, Bin Li

**Affiliations:** ^1^ Department of Social Medicine, Washington Institute for Health Sciences, Arlington, Virginia.; ^2^ Department of Neurosciences, Georgetown University Medical Center, Washington, District of Columbia.

**Keywords:** artificial intelligence, developing countries, healthcare, rural areas, service network

## Abstract

**Background:** Artificial intelligence (AI) is a rapidly developing computer technology that has begun to be widely used in the medical field to improve the professional level and efficiency of clinical work, in addition to avoiding medical errors. In developing countries, the inequality between urban and rural health services is a serious problem, of which the shortage of qualified healthcare providers is the major cause of the unavailability and low quality of healthcare in rural areas. Some studies have shown that the application of computer-assisted or AI medical techniques could improve healthcare outcomes in rural areas of developing countries. Therefore, the development of suitable medical AI technology for rural areas is worth discussing and probing.

**Methods:** This article reviews and discusses the literature concerning the prospects of medical AI technology, the inequity of healthcare, and the application of computer-assisted or AI medical techniques in rural areas of developing countries.

**Results:** Medical AI technology not only could improve physicians' efficiency and quality of medical services, but other health workers could also be trained to use this technique to compensate for the lack of physicians, thereby improving the availability of healthcare access and medical service quality. This article proposes a multilevel medical AI service network, including a frontline medical AI system (basic level), regional medical AI support centers (middle levels), and a national medical AI development center (top level).

**Conclusion:** The promotion of medical AI technology in rural areas of developing countries might be one means of alleviating the inequality between urban and rural health services. The establishment of a multilevel medical AI service network system may be a solution.

## Introduction

Artificial intelligence (AI) is a rapidly developing field of computer science that uses computers to simulate human learning, memory, analysis, and even innovation, which usually require human intelligence.^1–3^ The idea of AI has existed for a long time. Alan Turing first conceptualized AI in his 1950 article “Computing Machinery and Intelligence,” in which he introduced the ideas of machine learning, genetic algorithms, reinforcement learning, and the Turing test. AI was officially named at a Dartmouth College conference in 1956.^4^

In the healthcare field, AI technology was first applied as a medical diagnostic decision support (MDDS) system. Miller reviewed the progress of MDDS systems from 1954 to 1993.^5^ In 1961, Warner et al. developed a pioneer MDDS system that could be used in the diagnosis of congenital heart diseases.^6^ One of the first practically used MDDS systems for clinical diagnosis and simulation exercises was developed by de Dombal et al. at the University of Leeds, United Kingdom, in 1969.^7,8^ In the early 1970s, Shortliffe developed an expert system called MYCIN that was used to identify the organisms involved in infectious diseases and make suggestions for the use of antibiotics.^9^ By the 1990s, a large number of MDDS systems were developed, including internal, forensic, veterinary medicine, pathology, radiology, psychiatry, and so on.^6–10^ Recently, with the rapid development of computer, internet, advanced statistics, machine learning, and neural networks technology, in addition to the increase in handheld and wearable networked devices, such as smartphones and watches, AI technology is bringing revolutionary changes across the healthcare field. Although clinical work cannot be completely replaced by AI robot doctors in the foreseeable future, medical AI technology will play a huge role in electronic health records (EHRs), diagnosis, treatment protocol development, patient monitoring and care, personalized medicine, robotic surgery, and health system management.^11,12^

In this article, we first briefly introduce the prospect of medical AI technology, and then review the inequities and quality of healthcare in rural areas of developing countries. Next, we discuss the roles of medical AI-related technologies, such as computer-assisted diagnosis and mobile clinical decision support systems (mCDSS), on healthcare outcomes in rural areas of developing countries. Finally, we propose a multilevel medical AI service network aimed at improving the accessibility and quality of rural healthcare services in developing countries, and urge the development of medical AI technology that is suitable for rural use.

## The Prospect of Medical AI Technology

### AI in clinical decisions

With the rapid development of medical technology, new research information has been produced faster and faster. The amount of information within the medical literature doubles every 3 years. It is estimated that if a physician wanted to stay completely up-to-date, he/she should read 29 h per workday. As such, it is not possible to rely solely on humans to keep up with it.^13^ In addition, big data, including EHRs, “omic” data (e.g., genomics, metabolomics, and proteomics), and sociodemographic and lifestyle-related information would be of no use without comprehensive analysis. We would like to say that AI technology is the only solution.^3^ IBM Watson is one of the leading AI healthcare support systems that can assist doctors in making efficient decisions. With its machine learning and natural language processing capabilities, this system helps doctors review patients' EHRs and further search-related medical research publications and guidelines. A double-blind study compared the decisions made by a tumor board with the one made by the Watson Oncology system. The results showed that 90% of the recommendations made by the system were concordant with the ones made by the tumor board, but the system only took 40 sec to complete the process.^13,14^

### AI in EHRs

In 2009, the United States Department of Health and Human Services started to encourage the adoption of EHRs.^15^ However, the implementation process has been challenging. The major barriers include low satisfaction with the system, interoperability problems, and lagging adoption in solo practices and nonprimary care practices. Now EHR documentation has become one of the most time-consuming tasks in healthcare facilities.^16^ Deliberato et al. suggested that AI technology could help healthcare providers collect, store, reformat, and trace clinical data, as well as develop personalized assessments and plans.^17^

### AI in diagnosis

Diagnostic errors are a serious threat to healthcare quality and safety. It is estimated that the rate of outpatient diagnostic errors is 5.08% in the United States, which is ∼12 million adults every year. About half of these errors could potentially be harmful.^18^ AI technology has been utilized to improve the quality of medical diagnosis, especially in radiology, due to the large volumes of medical image data. A radiologist, Keith Dreyer at Harvard Medical School, claimed that “Meaningful AI will improve quality, efficiency, and outcomes.”^19^ Esteva et al. trained deep convolutional neural networks (CNN) based on a dataset of 129,450 clinical images to diagnose skin cancer. The results demonstrated that this system is able to classify skin cancer at a comparable level to dermatologists. They hypothesized that smartphones might be a low-cost method of helping to extend the reach of dermatologists to improve access to diagnostic care.^20^ Liu from Google, Inc. reported a CNN framework to aid the pathological diagnosis of breast cancer metastasis in lymph nodes. The results showed that this system could improve the speed, accuracy, and consistency of diagnosis, as well as reduce the false negative rate to a quarter of the rate experienced by human pathologists.^21^ In fact, AI technology has been widely introduced into various fields of diagnostics. For example, Dawes et al. recently published a magnetic resonance imaging-based algorithm of cardiac motion that allowed them to accurately predict the outcomes of patients with pulmonary hypertension.^22^ Moss et al. employed an automated rhythm classification methodology to analyze continuous electrocardiograms (ECGs) in critically ill patients, and concluded that AI technology was able to generate additional information and insights into data that might have been missed by physcians.^23^ Furthermore, Lee et al. reviewed promising results from recent studies using AI in stroke imaging, and suggested that AI technology may play a critical role in the management of stroke patients through an individualized plan.^2^

### AI in personalized medicine

Personalized medicine (precision medicine) is a new healthcare model, in which the treatment and prevention of diseases are based on individuals' conditions, including genetic information, psychosocial characteristics, environment, and lifestyles. All of this information will produce a vast amount of data, which can only be analyzed and integrated by AI technology. Mesko claimed that “There is no precision medicine without AI.”^24^ For example, DeepVariant, developed by Google, Inc., is a highly accurate genomic analysis system based on deep neural network technology.^25^

### AI in healthcare system management

Current healthcare systems focus on treatment-based care, which cannot provide appropriate low-cost interventions for healthy high-risk subjects. At the same time, the epidemic of chronic disease has caused a huge economic burden worldwide. In the United States, frustration with the healthcare system has led the government to consider introducing AI personal health monitoring platforms into healthcare management systems, to reduce rising healthcare costs and improve healthcare outcomes.^12,25,26^ AiCure, a smartphone program supported by the United States National Institutes of Health, has been developed to monitor patients' medications and conditions.^27^

Overuse of healthcare is a growing problem due to overdiagnosis caused by improvements in the sensitivity of medical detection methods. It is estimated that up to a third of cancer diagnoses detected through screening may reflect overdiagnosis and ∼30% of people diagnosed with asthma may not actually have the disease. Overtesting and overtreatment are also prevalent; for example, it was shown that over 50% of testing before cataract surgery was unnecessary and probably raised risks.^28^ Overprescription of antibiotics is also a very common problem, especially in developing countries, caused by insufficient training of health workers. Introduction of medical AI technology into healthcare management systems might help to identify unnecessary diagnoses and treatments.^29,30^

Therefore, medical AI technology not only focuses on the classical interactions between patients and healthcare providers, but it can also be used in the management of health systems for large-scale organizations. The systems can monitor health expenditures, cost recovery, and responses to treatment, thereby increasing population wellness and quality of care while decreasing costs.^1,31^

### AI in medical robots

The applications of medical AI technology also include assistive medical robots and devices. For example, telerobots can facilitate communication between patients with medical professionals; assistive walking devices can help with maneuvering, walking, standing, or sitting; and animal-like robots can communicate with and entertain patients.^32^ Robots can also be used in surgery as assistant surgeons. The da Vinci Surgical System is one of the most commonly used robotic surgical systems; over 3400 sets had been used by 2015.^33^

## The Inequities of Health Services in Rural Areas of Developing Countries

Achieving health equity and improving quality of healthcare for vulnerable populations are important social missions, especially in developing countries. Some governmental agencies, organizations, and academic institutions have paid attention to this issue and have sought solutions.^34,35^ Developing countries refer to countries in which the Gross National Income per capita per year does not exceed $11,905. Rural areas refer to areas in which the population density does not exceed 150 people per square kilometer.^36^ In developing countries, the life expectancies and health status of rural residents are generally worse than those of urban residents. Poverty is one of the biggest social determinants. Limited access to qualified healthcare is the immediate cause of poor health status. Low public health spending, low coverage of health insurance, a limited benefit package, shortages of health professionals and facilities, lack of training for health workers, transportation difficulties, and so on, all contribute to the low quality of healthcare in rural areas of developing countries.^36–38^

In developing countries, shortages of skilled healthcare providers in rural area are more severe than in developed countries. The reasons for this include a lack of students with a rural background, relatively low wages, poor working and living conditions, excessive workloads, lack of opportunities for continuing education and professional development, already existing shortages of health workers nationwide, and a larger proportion of rural populations in these countries.^36^ In developing countries, rural residents accounted for 68% (3.4 billion) of the total population in 2016, and this number will increase and reach its peak in the 2020s. On the other hand, rural residents accounted for only 19% of the total population in developed countries in 2016.^37,39^ About 90% of the global rural population live in Asia and Africa, and half of these (45%) live in India (857 million) and China (635 million). In Africa, Nigeria (95 million) and Ethiopia (78 million) have the largest rural populations. Regarding the doctor-to-population ratio, for India the density of doctors in urban areas was 1.71 per 1000 population, whereas that in rural areas was 0.45 per 1000 population.^40^ In China, the densities of doctors in urban and rural areas were 3.00 and 1.33 per 1000 population, respectively.^41^ The situation in Africa is the worst. In Nigeria, the densities of doctors in urban and rural areas were 0.14 and 0.01 per 1000 population, respectively, and in Ethiopia they were 0.07 and 0.02 per 1000 population, respectively.^42^

Due to the poor working environment, it is difficult to attract and retain high-quality healthcare providers in rural areas. To compensate for the shortage of physicians, many developing countries launch some abbreviated training programs for becoming a physician, or they authorize nurses to perform certain physician tasks. For example, there are many secondary medical vocational schools and junior medical colleges in China, in which students who graduated from middle or high school are given 3 years of medical training to become physicians. In 2014, around 52% of physicians (2.9 million) had less than a bachelor's degree, and most of them were working in rural areas of China. Although this can adequately meet the urgent demands for health workers in rural areas, the medical knowledge and skills of these doctors are insufficient.^43^

Even if physicians working in rural areas have sufficient knowledge and skills, they lack the support of other health providers (i.e., specialists, pharmacists, and laboratory technicians). In such situations, they generally have to provide a wider range of services. This professional isolation and varying scope of practice will reduce the quality of medical services.^36^ Today, medicine is a rapidly developing discipline. The expertise of rural physicians cannot be updated and improved due to the barriers to participating in continuing education and professional development.^44^

As a result, the quality of healthcare is low in rural areas of developing countries. Maternal mortality ratio (MMR) is widely accepted as a general indicator of healthcare quality within an area. According to the International Labor Organization, the global MMR is 29 deaths per 10,000 live births in rural areas, as compared with 11 in urban areas. In Asia and the Pacific region, the MMR in rural and urban areas were 18 and 8 deaths per 10,000 live births, respectively. The highest MMR is found in rural Africa, which was as high as 55 deaths per 10,000 live births, compared with 29 in urban areas. In contrast, the MMR in developed areas is very low, and there is no difference between urban and rural areas. For example, in North America the MMR in both urban and rural areas were 2 deaths per 10,000 live births.^37^

To address the inequities of health services in developing countries, the International Labor Organization recommended that governments should build universal health coverage and provide equitable effective access for rural populations, with the guiding principles of availability, affordability, and financial protection.^37^

## The Roles of Medical AI-Related Techniques in Rural Areas

As discussed above, many rural areas of developing countries have few trained physicians, and a large number of patients need to be treated by nurses or paramedical health workers. However, most problems were simple, repetitive, and treatable with a few safe, essential drugs. Computer-assisted medical technology was an early term used to refer to medical AI technology. In 1998, a computer-assisted diagnostic system, the Early Detection and Prevention System (EDPS), was developed in India for rural clinics without a physician. The system provided guidance and recommendations for nurses or experienced paramedical personnel. A study conducted by Kempegowda Institute of Medical Science in Bangalore, India showed that the overall rate of consistency between the EDPS and physicians was 94% for 933 patients. Another study found that patient responses were positive, as they believed the computer system was more accurate and had more in-depth interaction with them than health personnel they had met; additionally, the village health nurses were interested in using this system in their practice.^45^

mCDSS refers to any mobile electronic device, such as mobile phones, laptops, iPads, and wearable devices, which provide medical advice for health workers to improve the quality of healthcare. Adepoju et al. systematically reviewed the use of mCDSS in rural settings of sub-Saharan Africa. Although there was no evidence that the mCDSS could improve the overall quality of healthcare in sub-Saharan Africa, a few studies have shown beneficial effects, including significant improvement in healthcare outcomes and reduction of overprescription of antibiotics. They also found that the mCDSS could improve patient–provider relationships through increased trust and confidence, and that health workers believed the systems could improve their efficiency, competence, and self-confidence in their work.^46^ Olajubu et al. concluded that full-scale development of this system with proper implementation would help extend the availability of healthcare from urban cities to rural areas, thereby reducing the number of casualties in vulnerable areas of developing countries, especially in Sub-Saharan Africa.^47^

Recently, healthcare in China's rural areas has been benefiting from medical AI technology. According to a news report from South China Morning Post, a portable all-in-one diagnostic station (weighing 11 pounds), which can run 11 tests, including blood pressure, electrocardiographs, and routine urine and blood analyses, has been used in village healthcare settings. This device, which was developed by an internet healthcare company supported by the national rural healthcare program, can automatically upload results and medical records to an online data analysis system and generate a diagnosis for village health workers to review and reference. Several large-technology companies in China are also investing in AI-driven smart clinics for rural regions, such as AI-powered Chabot, which can communicate with patients, provide medical advice, and conduct online training for health workers in rural areas.^48^

In addition to the application of AI technology in primary health settings, many AI-driven systems have been developed for special diseases in rural areas. For example, a low-cost swallowable endoscopic capsule with AI analysis technology can be used to screen for upper gastrointestinal cancers, thus replacing expensive or difficult traditional screening equipment. This device is particularly suitable for rural areas of developing countries, where the majority of gastric cancer cases occur.^49^ Furthermore, acute leukemia is a malignant disease and its treatment effect depends on the identification of the leukemia type or subtype; however, advanced methods are very expensive and not available in most hospitals in developing countries. Escalante et al. reported a highly effective AI method to classify acute leukemia based on the morphological properties of bone marrow images. This method could provide an alternative to expensive diagnostic methods in developing countries.^50^ In addition, the complex nature of peripheral neuropathies causes a large portion of cases to remain undiagnosed in developing countries due to a lack of expert neurologists. Kunhimangalam et al. reported a novel AI clinical decision support system that may provide diagnostic and treatment medical opinions for peripheral neuropathies. Compared with experts' opinions, this system showed 93.3% accuracy. This system provides a solution for rural patients with peripheral neuropathies in the absence of specialists.^51^ Moreover, Oliveira et al. reported a new, automated, mobile device-based AI diagnostic system, which can analyze Giemsa-stained peripheral blood samples combined with light microscopy images to diagnose malaria. The accuracy of the system was 91% on average.^52^ The above examples suggest that accessibility barriers of rural areas in developing countries can be addressed through low-cost diagnostic tools that replace more expensive or difficult traditional screening equipment that is not available in rural areas.

Thus, medical AI technology can not only improve doctors' efficiency and the quality of healthcare services, and reduce medical costs, but nurses and paramedical health workers can also be trained to use this tool to compensate for a lack of doctors. However, having only a few medical AI devices, such as an mCDSS or a portable all-in-one diagnostic station, is far from sufficient. To improve the overall level of healthcare services in rural areas of developing countries, an effective medical AI system requires a series of supports, such as infrastructure (electricity and internet), continuous training, supervision, financial support, technical upgrades, and supportive public health policies.^46^ Therefore, in this article, we propose a comprehensive multilevel medical AI service network aimed to improve the availability and quality of healthcare services in rural areas of developing countries.

## A Vision of Medical AI Technology for Rural Areas of Developing Countries

### A multilevel medical AI service network

Regarding the situation of rural areas, the medical AI system should be specifically designed for rural areas. We hereby describe the multilevel medical AI service network aimed at achieving this goal.

(1) Basic level—frontline medical AI system: This system will be used in the most basic level of rural healthcare settings, such as village or personal clinics. The poor general economic conditions, inconvenient transportation, lack of or unstable communication and electric power facilities, inadequate training of medical workers, and relatively simple diseases require this system to have the following features: (a) An economical and practical clinical decision support system, which mainly focuses on common diseases. For complicated or serious diseases, only referrals can be prompted; (b) affordability; the cost should be limited to between $500 and $1000; (c) compact and solid structure; it is easy to carry or drag and may be equipped with shock, sand, moisture, and waterproof measures; (d) connectivity; equipped with a variety of networking methods for information transmission and system upgrades (e.g., telephone line, wireless, cable); (e) equipped with rechargeable battery, or even a hand-cranked generator; and (f) a friendly operation interface that is easy to learn and requires minimal training.^36,45^ In general, the main configuration of this system includes a laptop and a portable diagnostic instrument. The diagnostic instruments can perform simple medical diagnoses, such as blood, urine, ECG, and other routine examinations. The laptop is installed with a combination AI medical program, including EHRs, diagnosis analysis, and advice systems.
(2) Middle levels—regional medical AI support centers: These could be set up in county hospitals and in state or provincial hospitals. The main roles of these include training primary health workers; maintaining, repairing, and upgrading the frontline medical AI systems; and collecting and reporting the epidemiological information received from primary EHRs. Additionally, these hospitals could be equipped with special medical AI systems to treat patients with serious and complicated illnesses.
(3) Top level—national medical AI development center: Its role is to coordinate the development, promotion, and upgrades of medical AI systems nationwide, and to foster international cooperation. To ensure the success of this multilevel medical AI service network, multiparty cooperation is needed. The governmental agents are responsible for providing funds and management; nonprofit organizations and charities can help raise funds; university and medical research institutes can design appropriate medical AI systems; medical equipment companies will be responsible for making products; and, finally, the local health agencies and medical organizations will be responsible for promoting the system. Continuous collaboration is critical to keep this system updated and follow the latest medical progress.

### Challenges and countermeasures

#### Financial issue

Their backward economic situation has always been the biggest problem for developing countries, especially in rural areas. Dalaba et al. studied the costs associated with implementation of a computer-assisted clinical decision support system for antenatal and delivery care in Northern Ghana. They observed a decrease in the proportion of complications during delivery and a reduction in the number of maternal deaths. The total financial cost per health worker trained was approximately $1060, of which the equipment cost (laptops, computer desks, and chairs) accounted for the largest proportion of the financial cost of 34% ($360). Personnel cost (technical officer and health worker) accounted for 28.6%, meeting and training costs accounted for 12.1%, transportation cost accounted for 8.5%, and all other costs accounted for 16.8%. Although this is not an issue for developed countries at all, it may represent a heavy burden for poor rural areas.^53^ Therefore, local governments and international organizations should be urged to strengthen financial support, while minimizing the costs. Increased affordability of computer hardware may foster these efforts.

#### Infrastructure issues

Electricity and/or the internet are unavailable, the transportation facilities are very inconvenient, and the natural environment is harsh in some rural areas. Therefore, we must address these issues during the system designing process.

#### Technical issues

In many developing countries, a frontline rural clinic probably has a nurse with minimal training, along with a paramedic or technician with a 10th or 12th-grade education. However, almost all medical AI systems have been developed to provide support for trained doctors.^45^ Thus, a specially designed friendly operating system, which is adapted for local rural health workers, is absolutely necessary. The increased workload was a frequently reported barrier to using medical AI-related technologies,^46^ so it is necessary to use an automatic and intelligent method of reducing workloads, such as voice recognition technology.

#### Training and professional issues

Multiple studies have revealed that initial and refresher training, as well as technical and supervisory support, were critical for the effective use of medical AI devices. In addition, the need for technical training was greater in older health workers with low computer literacy. Reports also showed that health workers sometimes disagreed with recommendations provided by the medical AI devices.^46^ Therefore, regional medical AI support centers must regularly conduct training and assessment for primary health workers to avoid misapplication of AI systems. At the same time, professional supports are needed when health workers feel conflict and uncertainty regarding medical AI systems. It was reported that health workers preferred to use medical AI systems when they realized the systems were based on updated best practices from a trusted national or international source.^46^ Therefore, regular system upgrades are also a key factor in promoting use and improving the medical service level.

#### Professionalism issues

Some worry that medical AI technology could place some doctors at risk for skill erosion in terms of diagnostic expertise and critical thinking, or it may put some doctors out of work or reduce their compensation. We do not think this will be a problem because current medical AI technology only provides advice for doctors; it does not replace the doctors' work. On the other hand, this system can only promote clinical skills for undertrained health workers. In rural areas, there are very few doctors; therefore, they are unlikely to lose their jobs or have their compensation reduced in the foreseeable future.^10^

#### Patient–provider relationships

Although a few reports have shown that medical AI-related technologies (e.g., mCDSS with a tablet) could decrease the effectiveness of patient consultation, such as missing nonverbal cues when concentrating on the tablet,^54^ most reports have shown improvements in trust between patients and providers.^45,46^ In short, the promotion of medical AI technology in rural areas of developing countries will have some challenges, especially financial issues, but these are not insurmountable.

## Conclusion

Medical AI technology has the potential to both improve the availability of healthcare access and healthcare quality within rural areas of developing countries. To realize this goal, we propose a multilevel medical AI service network, which includes a frontline medical AI system (the first level), regional medical AI support centers (the middle levels), and a national medical AI development center (the highest level). We recommend that governments, nonprofit organizations, charities, university research institutes, and AI and medical equipment companies cooperate continuously to develop, promote, maintain, and upgrade the system. In addition, the government health sector could have a permanent agency tasked with managing this project. We hope this review can provide a reference for international organizations (e.g., WHO, International Labor Organization), national government health departments, and other related organizations to improve healthcare for patients in developing nations.

## Abbreviations Used

AI: artificial intelligence
CNN: convolutional neural networks
ECGs: electrocardiograms
EDPS: Early Detection and Prevention System
EHRs: electronic health records
mCDSS: mobile clinical decision support systems
MDDS: medical diagnostic decision support
MMR: maternal mortality ratio
